# 7-year outcomes in diabetic patients after coronary artery bypass graft in a developing country

**DOI:** 10.1186/s12872-023-03279-8

**Published:** 2023-05-12

**Authors:** Parmida Sadat Pezeshki, Farzad Masoudkabir, Mina Pashang, Ali Vasheghani-Farahani, Arash Jalali, Saeed Sadeghian, Kaveh Hosseini, Soheil Mansourian, Shahram Momtahan, Abbasali Karimi

**Affiliations:** 1 Students’ Scientific Research Center (SSRC), Tehran University of Medical Sciences, Tehran, Iran; 2 Cardiac Primary Prevention Research Center, Cardiovascular Diseases Research Institute, Tehran University of Medical Sciences, Tehran, Iran; 3 Tehran Heart Center, Cardiovascular Diseases Research Institute, Tehran University of Medical Sciences, Tehran, Iran; 4 Research Center for Advanced Technologies in Cardiovascular Medicine, Cardiovascular Diseases Research Institute, Tehran University of Medical Sciences, Tehran, Iran; 5 Cardiovascular Diseases Research Institute, Tehran Heart Center, Kargar Street, Jalal al-Ahmad Crossroads, Tehran, 1411713138 Iran

**Keywords:** Diabetes, Coronary artery bypass graft surgery, Major adverse cardiac and cerebrovascular events, Acute coronary syndrome, Revascularization

## Abstract

**Background:**

Revascularization in diabetic patients with coronary artery disease remains a challenge in cardiology practice. Although clinical trials have reported the mid-term superiority of coronary artery bypass grafting (CABG) surgery over percutaneous coronary intervention in these patients, little is known about the long-term outcomes of CABG in diabetic patients compared to non-diabetics, particularly in developing countries.

**Methods:**

Between 2007 and 2016, we recruited all patients who underwent isolated CABG in a tertiary care cardiovascular center in a developing country. The patients were followed at 3–6 months and 12 months after surgery, and then annually. The study endpoints were 7-year all-cause mortality and major adverse cardiac and cerebrovascular events (MACCE).

**Results:**

Of 23,873 patients (17,529 males, mean age 65.67 years) who underwent CABG, 9227 (38.65%) patients were diagnosed with diabetes. After adjustment for potential confounders, patients with diabetes experienced a 31% increase in MACCE seven years after surgery compared to the non-diabetic patients (HR = 1.31, 95% CI: 1.25–1.38, P-value < 0.0001). Meanwhile, diabetes contributes to a 52% increase in the risk of all-cause mortality after CABG (HR = 1.52, 95% CI: 1.42–1.61, P-value < 0.0001).

**Conclusions:**

Our study showed a higher risk of all-cause mortality and MACCE at seven years in diabetic patients undergoing isolated CABG. The outcomes in the studied center in a developing country were comparable to western centers. The high incidence of adverse outcomes in the long term in diabetic patients implies that not only short-term but long-term measures should be taken to improve the CABG outcomes in this challenging patient population.

**Supplementary Information:**

The online version contains supplementary material available at 10.1186/s12872-023-03279-8.

## Introduction

Diabetes significantly increases the risk of developing cardiovascular diseases, including coronary artery disease (CAD). The mortality risk due to CAD has been about three times higher in diabetic patients than in patients without diabetes [1]. Moreover, there is a higher tendency for developing multi-vessel disease (MVD) in diabetic patients, which further poses some considerable challenges for the selection of the revascularization therapy and optimization of the perioperative and post-operative outcomes in this exigent group of patients [2].

Although better outcomes after coronary artery bypass graft (CABG) surgery in diabetic patients with MVD rather than after balloon angioplasty have been long postulated [3], investigations on whether CABG is still the optimal treatment for diabetic patients in comparison with the percutaneous coronary intervention (PCI) with the administration of the drug-eluting stents were performed. For instance, considering the multi-center Future Revascularization Evaluation in Patients with Diabetes Mellitus: Optimal Management of Multivessel Disease (FREEDOM) trial results CABG is suggested over PCI as the treatment of choice for diabetic patients [4, 5]. The result of this pillar study demonstrated a significantly lower incidence of adverse events in patients undergoing CABG in a follow-up period of more than five years [6]. In agreement with these results, it has been reported that most diabetic patients with 3-vessel CAD undergo CABG (although this is not the case for patients with the 2-vessel disease.) [7].

In the last couple of decades, the incidence, guidelines and treatments, and subsequently mortality and comorbidities of diabetes, have been relatively changed [8–10]. Moreover, the outcomes of CABG could have been influenced by various factors throughout the years, such as the administration of the left internal mammary artery (LIMA) versus Saphenous vein graft (SVG) or other grafts as the conduit [11]. Also, the growing tendency to undergo medical treatment or intervention instead of surgery led to a drop in surgical trends even among the indicated patients for CABG [12]. For instance, although, according to a study in the united states, the post-operative mortality of CABG has remained unchanged over the years, the patients undergoing surgery have become older and with more comorbid conditions such as diabetes [13]. Diabetes has been associated with higher post-surgery mortality, especially in patients more aged than 80 years old [14].

This trend has to be studied in developing countries, too. Considering all of these trends, the evaluation of how they have affected the CABG outcomes in diabetic patients, whether the survival is still worse, and how much the incidence of adverse long-term outcomes is higher in patients with diabetes compared to the non-diabetic patients are among the most critical issues that should be addressed. The increasing number of diabetic patients undergoing CABG necessitates studies like ours, which evaluate the short-term or long-term outcomes in this group of patients. Consequently, in the present study, we sought to assess the mortality rate and major adverse cardio-cerebrovascular events (MACCE), including the incidence of all-cause mortality, the repetition of revascularization (either PCI or a second CABG), cerebrovascular accident (CVA), and acute coronary syndrome (ACS) in 23,800 patients that underwent CABG in a median follow-up period of 7 years.

## Methods

### Study population

We used the data of 23,873 patients from the single-center-based cohort of CABG Follow-up Registry of Tehran Heart Center who underwent CABG between 2007 and 2016. Patients were separated into two groups of diabetic (N = 9227) and non-diabetic (N = 14,624) patients. Patients were considered diabetic if they had a positive history of self-reported diabetes, confirmed diagnosis of diabetes in their medical records, and/or if they were on anti-glycaemic agents. Patients without any previous criteria who had 2 fasting blood glucose measurements > = 126 mg/dL, in their routine pre-operative blood tests, were also considered diabetic.

Based on the 2011 ACCF/AHA guidelines [11], CABG was performed on patients with high-grade blockages in any of the major coronary arteries, such as a > 50% blockage in the left main coronary artery or left circumflex coronary artery (left-main disease), blockage of two coronary arteries (including left anterior descending (LAD) artery involvement, and three-vessel involvement with > 70% blockage, or who had failed PCI to remove the blockages. Our study protocol complied with The ethical guidelines of the 1975 Declaration of Helsinki and was conducted under the approval of the ethics committee of Tehran Heart Center. We obtained verbal informed consent from all the enrolled patients.

### Follow-up protocol

The follow-up protocol constituted in-person visits 4–6 months and one year after surgery, and then annually. The patients who couldn’t take part in direct clinic visits were followed by telephone interviews. We documented and registered the patients’ demographic characteristics, risk factors for cardiovascular diseases (CVD) (i.e., hypertension, dyslipidemia, family history of premature coronary artery disease, cigarette smoking, and opium consumption), laboratory findings including glucose and lipid profile and the serum creatinine levels in each visit. Moreover, the incidence of major adverse cardio-cerebrovascular events (MACCEs; defined as a composite of all-cause mortality, ACS, stroke, or transient ischemic attack, and the need for repeat revascularization (percutaneous coronary intervention or redo-CABG)) was evaluated in each visit. A family history of premature coronary artery disease was described as a history of coronary artery disease, including acute myocardial infarction or documented coronary artery disease (through invasive coronary angiography or computed tomography coronary angiography) in a male or female first-degree relative under 55 or 65 years old, respectively.

### Study endpoints

The primary endpoint in our study was the composite endpoint of MACCE constituting all-cause mortality, ACS, stroke or transient ischemic attack, and the need for repeat revascularization. The secondary endpoint was all-cause mortality.

### Statistical methods

The normally distributed variables were reported as mean with standard deviation (SD) and, using the student’s t-test, were compared between the two groups of diabetic and non-diabetic patients. We described the skewed distributed variables as median with interquartile range boundaries, and by applying the Wilcoxon-Mann-Whitney test, we compared them between the two above-mentioned groups. Histogram charts, as well as descriptive measures of central tendency and dispersion normality, were administered to check the normality of the variables. Categorical variables were compared between the two groups by applying the chi-squared test. Both the unadjusted and adjusted effects of diabetes status on mortality and MACCEs were assessed by using the Cox proportional hazards model, and the results were expressed through hazard ratio (HR) along with 95% confidence intervals (CIs). The covariates, which were identified as independent risk factors for the mortality and MACCE after surgery which were not evenly distributed among the two groups of diabetes status with P values less than 0.05, including age, sex, hypertension, body mass index (BMI), dyslipidemia, off-pump surgery, graft number, total hours spent in the intensive care unit (ICU) and under ventilation, creatinine level, cigarette smoking, and opium consumption, were considered as potential confounding factors (Additional File Table 1). The number of each component of MACCEs, including ACS, revascularization, and cerebrovascular accident in both diabetic and non-diabetic patients, were reported.

Statistical analyses were conducted using IBM SPSS Statistics for Windows, version 23.0 (IBM Corp., Armonk, NY, USA). Competing risk analyses were done by applying the ‘stcrreg’ module in Stata software version 14.2.

## Results

### Population

The baseline characteristics of our study population are summarized in Table 1. In our study population of 23,851 patients undergoing CABG, 9,227 patients (38.7%) had diabetes, and 14,624 patients (61.3%) had no diabetes at the time of surgery. The group of diabetic patients was significantly older and had a higher BMI and a lower ejection fraction (EF). The proportion of males was higher in non-diabetic patients than the diabetics. Diabetic patients were more likely to have hypertension or dyslipidemia. The number of current or former cigarette smokers in patients with diabetes was similar to the other patients without diabetes. In contrast, the number of opium ever-users was significantly lower in diabetic patients. Also, Heart failure with reduced ejection fraction (HFrEF) was more prevalent in diabetic patients.

**Table 1:** Baseline characteristics of the study population based on the status of diabetes

	Total(n = 23,851)	Diabetic(n = 9,227)	Non-Diabetic(n = 14,624)	P-value
**Pre-operative variables**				
Age	65.67 (10.20)	65.98 (9.28)	65.48 (10.74)	< 0.0001
Male Gender	17,529 (73.5%)	5709 (61.9%)	11,820 (80.8%)	< 0.0001
BMI	27.28 (4.28)	27.63 (4.37)	27.05 (4.21)	< 0.0001
Hypertension	12,688 (53.2%)	5747 (62.3%)	6941 (47.5%)	< 0.0001
Dyslipidemia	13,322 (55.9%)	6216 (67.4%)	7106 (48.6%)	< 0.0001
PreMI u7	1875 (7.9%)	709 (7.7%)	1166 (8.0%)	0.417
PreMI u24	627 (2.6%)	228 (2.5%)	399 (2.7%)	0.225
Creatinine	0.97 (0.80, 1.19)	0.90 (0.78, 1.12)	1.00 (0.80, 1.20)	< 0.0001
Family history	8974 (37.6%)	3513 (38.1%)	5461 (37.4%)	0.265
Cigarette smoking:				< 0.0001
1-Current	4163 (17.5%)	1136 (12.4%)	3027 (20.7%)	
2-Former†	4429 (18.6%)	1480 (16.1%)	2949 (20.2%)	
Left main disease*	1973 (8.3%)	716 (7.8%)	1257 (8.7%)	0.022
Opium consumption***	3626 (15.3%)	1138 (12.4%)	2488 (17.1%)	< 0.0001
COPD	875 (3.7%)	338 (3.7%)	537 (3.7%)	0.998
Total EF	46.84 (9.36)	46.13 (9.53)	47.29 (9.21)	< 0.0001
HFrEF	6855 (28.7%)	2886 (31.3%)	3969 (27.1%)	< 0.0001
**Intra-operative variables**				
Off-pump	1716 (7.3%)	723 (7.9%)	993 (6.9%)	0.003
Graft number	3.00 (3.00, 4.00)	4.00 (3.00, 4.00)	3.00 (3.00, 4.00)	< 0.0001
**Post-operative variables**				
Length of hospital stay (days)	7.00 (6.00, 8.00)	7.00 (6.00, 9.00)	6.00 (6.00, 8.00)	< 0.0001
Total ICU hours	28.00 (23.00, 65.50)	29.00 (23.00, 67.30)	28.00 (22.50, 63.50)	< 0.0001
Total ventilation hours**	9.50 (7.30, 12.50)	10.00 (7.50, 12.50)	9.30 (7.00, 12.30)	< 0.0001

BMI: body mass index; MI: myocardial infarction; COPD: chronic obstructive pulmonary disease; EF: ejection fraction; HFrEF: Heart failure with reduced ejection fraction. PreMI u7: previous MI (within 7 days of surgery); PreMI u24: previous MI (within 24 h of surgery)

†Ever smokers who didn’t smoke one or a part of a cigarette within 30 days of the investigation

*Left main disease was defined as an obstruction > 50% in one of 2 major left circulation branches (left anterior descending or left circumflex coronary artery)

**Ventilation hours were assessed in a subgroup of patients that underwent on-pump surgery

***Number of opium-ever users, including former and current opium consumers. This includes the number of recreational opium users as well

Continuous variables are presented as mean (SD) or median (25th, 75th percentiles)

Categorical variables are described as frequency (percentage); n (%)

### Follow up

The follow-up period in our group of patients ranges from 2.0 to 169 months. The median follow-up period calculated by the inverse Kaplan-Meier curve was about seven years or 84.7 months (95% confidence interval: 83.5–85.9 months). Only 211 patients (0.9%) were completely lost to follow up. The outcomes were evaluated at the time of 7-year post-surgery.

### Endpoints

**Fig. 1:**
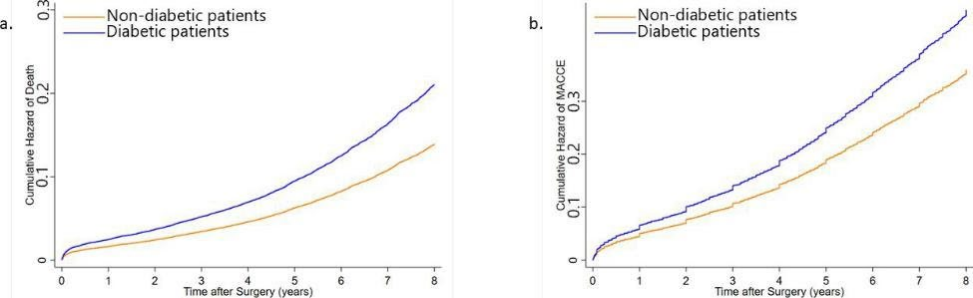
Adjusted cumulative hazard of death and major adverse cardiac and cerebrovascular events (MACCE). Adjusted cumulative hazard of death (a) and major adverse cardiac and cerebrovascular events (MACCE) (b) after coronary artery bypass grafting (CABG) surgery according to the diabetes status is analyzed using the cox proportional hazards model and depicted

**Fig. 2:**
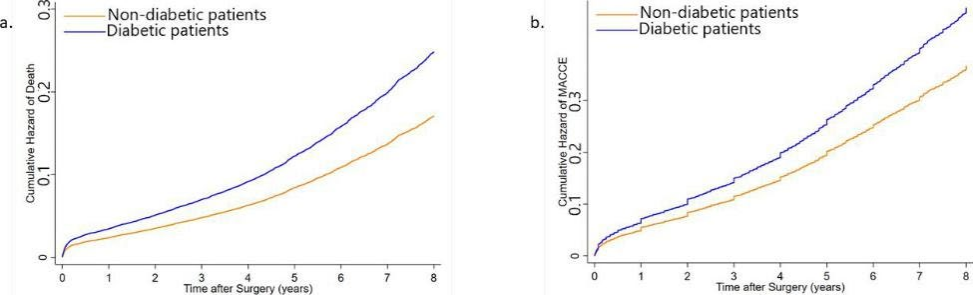
Adjusted cumulative hazard of death and major adverse cardiac and cerebrovascular events (MACCE). The Adjusted cumulative hazard of death (a) and major adverse cardiac and cerebrovascular events (MACCE) (b) after coronary artery bypass grafting (CABG) surgery according to the diabetes status by applying the inverse probability weighting (IPW) model is calculated and demonstrated

The 7-year cumulative incidence of all-cause mortality was 19.46% (1796 patients) and 14.12% (2065 patients) in diabetic and non-diabetic patients, respectively. (HR 1.59, 95% CI: 1.50–1.68, P-value < 0.0001) (Table 2). After adjustment for the confounding variables, the HR of diabetes for death remained significant. Diabetes attributes to a 52% increase in the risk of mortality after CABG. (HR 1.52, 95% CI 1.42–1.61, P-value < 0.0001) (Fig. 1a.) The inverse probability weighting (IPW) analysis results were similar to that found in the Cox proportional hazards model. (HR 1.52, 95% CI 1.42–1.61, P-value < 0.0001) (Fig. 2a.) In the univariate analysis, all the other investigated variables except for cigarette smoking and graft number were shown to increase the risk for all-cause mortality significantly (Additional File Table 1). However, after adjustment for the rest of the variables, BMI, under-24-hours post-surgery myocardial infarction, and graft number were the variables that did not significantly affect the all-cause mortality rate. Meanwhile, the effect of all the other variables was depicted to be independently significant (Additional File Table 3).

**Table 2:** Effect of diabetes on MACCE and all-cause mortality after CABG

	MACCE			All-cause mortality				
	**HR**	**95% CI**	**P-value**		**HR**	**95% CI**	**P-value**	
Unadjusted	1.43	1.36–1.49	< 0.0001		1.59	1.50–1.68	< 0.0001	
Adjusted	1.31	1.25–1.38	< 0.0001		1.52	1.42–1.61	< 0.0001	
IPW	1.31	1.24–1.37	< 0.0001		1.45	1.36–1.55	< 0.0001	

Note: in the data presented as adjusted HR, the adjustment was performed for age, sex, hypertension, body mass index (BMI), dyslipidemia, off-pump surgery, graft number, total hours spent in the intensive care unit (ICU) and under ventilation, creatinine level, cigarette smoking, and opium consumption

MACCE: major adverse cardiac and cerebrovascular events; CABG: coronary artery bypass grafting; IPW: inverse probability weighting; HR: hazard ratio in adjusted and IPW sections represents the independent effect of diabetes on MACCE and all-cause mortality after adjustment for the rest of the assessed covariates by using the Cox proportional hazards model and inverse probability weighting model, respectively; CI: confidence interval

The other endpoint of our study was a composite MACCE endpoint of ACS, CVA, the need for a second revascularization therapy, and death. The incidence of each MACCE component in all of the patients and in the two groups of diabetic and non-diabetic patients is described in Table 3. Death was the most prevalent MACCE, with ACS and CVA being the second and third most prevalent MACCE in our group of patients, respectively. The excess risk of diabetes was significant for MACCE both before (HR 1.43, 95% CI 1.36–1.49, P-value < 0.0001) and after adjustment (HR 1.31, 95%CI 1.25–1.38, P-value < 0.0001) (Fig. 1b.) for competing confounding factors, and also in IPW analysis (HR 1.31, 95% CI 1.24–1.37, P-value < 0.0001) (Fig. 2b.). (Table 2) In the univariate analysis for the other competing variables, BMI, dyslipidemia, under-24-hour post-surgery myocardial infarction, and smoking were not appeared to increase the risk of MACCE (Additional File Table 3). After adjustment, all of the evaluated variables, except for BMI, under-24-hour post-surgery myocardial infarction, and HFrEF, were identified as independent risk factors for MACCE. Dyslipidemia and smoking which were not statistically significant risk factors for MACCE in the univariate analysis were demonstrated to be independently associated with MACCE following adjustment for the rest of the variables (Additional File Table 4).

**Table 3:** The number of each MACCE component in the study population based on the status of diabetes after CABG.

MACCE component	Total(n = 23,851)	Diabetic(n = 9,227)	Non-Diabetic(n = 14,624)	P-value
ACS	2977(12.48)	1202(13.03)	1775(12.14)	0.043
CVA	765(3.21)	372(4.03)	393(2.69)	< 0.0001
Revascularization	90(0.38)	37(0.40)	53(0.36)	0.636
Death	3861(16.19)	1796(19.46)	2065(14.12)	< 0.0001

MACCE: major adverse cardiac and cerebrovascular events; CABG: coronary artery bypass grafting; ACS: acute coronary syndrome; CVA: cerebrovascular accident

Components are described as frequency (percentage); n (%) in total patients and in diabetic and non-diabetic patients, respectively

## Discussion

In our study on a retrospective cohort including 23,851 patients who underwent CABG in a period of 10 years between 2007 and 2017, about 39% of patients had diabetes at the time of surgery. The main finding of our study was that compared to non-diabetic patients, diabetic patients encountered a 52% and 31% increase in the risk of 7-year mortality and MACCE after CABG, respectively. Diabetic patients had a higher length of stay for their hospitalization, have spent longer hours in ICU, needed longer hours of ventilation, and were more likely to have MVD and a higher number of grafts. The group of diabetic patients was significantly older, and had a higher body mass index (BMI) and a lower EF. However, these differences could primarily depict statistical significance due to the large number of enrolled patients rather than implying clinical importance.

Since diabetic patients constitute a major and demanding proportion of the patients undergoing CABG, evaluating the outcomes and proposing solutions to improve these outcomes are mandatory. Additionally, diabetes is a condition accompanied by chronic inflammation and macro- and microvascular dysfunction. Some of the most significant complications of it, such as chronic kidney disease and myocardial infarction, would not appear until after a long course of time. Hence, the follow-up and investigation of the long-term post-operative outcomes in a large group of patients would be worth evaluation.

Studies in the past two decades intended to investigate the correlation of diabetes with short-term or long-term outcomes of CABG. Our study was a single-center study in Tehran Heart Center, an educational, tertiary care cardiovascular center in Iran. We sought to compare the post-CABG survival and long-term outcomes of diabetic and non-diabetic patients from a developing country with the ones reported from Western countries. In one of the substantial studies in the United states, recruiting 2,278 patients with diabetes and 9,920 patients without diabetes, diabetic patients had significantly lower 5-year and 10-year survival. This study did not report a composite outcome as MACCE. However, they claimed that the difference between the two groups in the long-term incidence of myocardial infarction and redo CABG surgery remained insignificant, whereas diabetic patients experienced a higher risk of post-CABG angioplasty in the period of 10 years [15]. These results are comparable to ours, as the incidence of revascularization (CABG or PCI) was not significantly different among the diabetic and non-diabetic patients included in our study. However, the incidence of ACS was significantly higher in diabetic patients. A study from Iceland looking at patients with or without diabetes undergoing isolated CABG with a median follow-up of 8.5 years revealed similar results to our study, as well [16]. Similar to our study, in this study, the composite endpoint of MACCE included mortality, stroke, myocardial infarction, and revascularization. Diabetes independently increased the risk of long-term MACCE by 40% (HR = 1.40, 95%CI: 1.17–1.67, adjustment for: sex, age, smoking, BMI, hypertension, and left ventricular EF). This HR is slightly higher than the HR of 1.31 reported in our study and it might be explained by the higher number of confounding factors that we did the adjustment for, in order to obtain the HR. One study from Canada found cardiac-specific survival at 5 and 10 years was lower in insulin-dependent patients with DM compared with both non-diabetic patients and patients with non-insulin-dependent DM [17]. Finally, Another Canadian study found Ten-year survival (p = 0.006) and survival free of major adverse cardiac events (p = 0.02) was decreased in the diabetic group [18]. In our study, a relatively considerable proportion of patients (39%) were diagnosed with diabetes, which renders the results more representative and comparable between the two groups of diabetic and non-diabetic patients. On that account, we believe these demonstrations can further be administered to improve the CABG long-term post-operative outcomes in patients with diabetes.

Higher levels of inflammatory factors such as TNF-α, IL-6, IL-18, and CRP, along with the traditional cardiovascular risk factors such as the duration of diabetes and higher prevalence of hypertension and dyslipidemia, have been suggested as some of the potential reasons for higher risk of MACCE in 30 days after surgery [19]. Another study in the United Kingdom failed to depict diabetes as an independent risk factor of in-hospital mortality. On the other hand, diabetes was significantly associated with short-term renal, neurologic, and gastrointestinal complications [20]. These studies have manifested the role of diabetes and its underlying pathological mechanisms, such as an enhanced inflammatory state, as risk factors for worse short-term outcomes. Diabetes is yet associated with some chronic conditions with gradual progression. Hence the assessment of long-term outcomes is worthy of note.

Regarding the increased risk of MACCE and all-cause mortality in our study, we assume that identifying pre-operative markers of poorer CABG outcomes in diabetic patients would be of benefit. Parameters such as higher preoperative HbA1C have been proposed as prognostic predictors of higher mortality and other post-operative complications (i.e., myocardial infarction, renal failure, or stroke) in diabetic patients [21–23]. Hence, glycemic control can improve the outcomes in this group of patients [24–26]. Still, there is a need for predictors of better prognosis and stratification methods to precisely identify the diabetic patients who would benefit the most from the surgery.

The significant strength of the present study is a large number of enrolled patients in addition to a relatively long follow-up period. Moreover, the successful follow-up rate of 99.1% was a big plus for our study. Additionally, the appropriate proportion of diabetic to non-diabetic patients compared to similar studies renders the results substantially comparable and reliable.

## Conclusions

Our study manifested a significantly higher risk of 7-year post-operative all-cause mortality and MACCE in diabetic patients compared to non-diabetic patients after CABG. We also evaluated and reported the differences in the incidence of traditional cardiovascular risk factors in these two groups of patients. Due to the higher incidence of undesirable outcomes in diabetic patients, also short-term and long-term actions should be taken to manage and improve the CABG outcomes in this demanding group of patients.

## Electronic supplementary material

Below is the link to the electronic supplementary material.

Additional File Table 1: The univariate all-cause mortality analysis. Additional File Table 2: The univariate MACCE analysis. Additional File Table 3: Independent effect of each variable on all-cause mortality after adjustment for the rest of the listed variables. Additional File Table 4: Independent effect of each variable on MACCE after adjustment for the rest of the listed variables.

## Data Availability

The datasets used and/or analyzed during the current study are available from the corresponding author upon reasonable request.

## Abbreviations

ACS: Acute coronary syndrome
BMI: Body mass index
CVD: Cardiovascular diseases
CVA: Cerebrovascular accident
CI: Confidence interval
CABG: Coronary artery bypass grafting
CAD: Coronary artery disease
EF: Ejection fraction
HR: Hazard ratio
HFrEF: Heart failure with reduced ejection fraction
ICU: Intensive care unit
IPW: Inverse probability weighting
LIMA: Left internal mammary artery
MACCE: Major adverse cardiac and cerebrovascular events
MVD: Multi-vessel disease
PCI: Percutaneous coronary intervention
SVG: Saphenous vein graft
SD: Standard deviation
