# Supplementary material for: 7-year outcomes in diabetic patients after coronary artery bypass graft in a developing country
Source: BMC Cardiovasc Disord. 2023 May 12;23:248. doi: 10.1186/s12872-023-03279-8 (PMC10182602; doi:10.1186/s12872-023-03279-8)
Supplement: Supplementary file 1 — Additional File Table 1: The univariate all-cause mortality analysis. Additional File Table 2: The univariate MACCE analysis. Additional File Table 3: Independent effect of each variable on all-cause mortality after adjustment for the rest of the listed variables. Additional File Table 4: Independent effect of each variable on MACCE after adjustment for the rest of the listed variables. [file 12872_2023_3279_MOESM1_ESM.docx]

**Supplementary tables:**

Supplementary table 1. The univariate all-cause mortality analysis.

|  | HR | 95% CI | P-value |
| --- | --- | --- | --- |
| Diabetes | 1.59 | 1.50-1.68 | <0.0001 |
| Age | 1.056 | 1.053-1.059 | <0.0001 |
| Male Gender | 0.930 | 0.872-0.992 | 0.027 |
| BMI | 0.983 | 0.976-0.990 | <0.0001 |
| Hypertension | 1.393 | 1.314-1.478 | <0.0001 |
| Dyslipidemia | 0.841 | 0.794-0.892 | <0.0001 |
| Off-pump | 1.715 | 1.517-1.938 | <0.0001 |
| PreMI u7 | 1.238 | 1.112-1.378 | <0.0001 |
| PreMI u24 | 1.202 | 1.010-1.430 | 0.038 |
| Creatinine | 1.308 | 1.274-1.343 | <0.0001 |
| Family history | 0.697 | 0.656-0.742 | <0.0001 |
| Cigarette smoking: |  |  |  |
| 1-Current | 0.999 | 0.921-1.083 | 0.980 |
| 2-Former | 1.064 | 0.988-1.145 | 0.099 |
| Left main disease* | 1.383 | 1.250-1.530 | <0.0001 |
| Graft number | 0.974 | 0.944-1.006 | 0.115 |
| Total ICU hours | 1.001 | 1.001-1.001 | <0.0001 |
| Total ventilation hours** | 1.001 | 1.001-1.001 | <0.0001 |
| Opium consumption*** | 1.238 | 1.145-1.339 | <0.0001 |
| COPD | 1.729 | 1.527-1.959 | <0.0001 |
| Total EF | 0.957 | 0.954-0.960 | <0.0001 |
| HFrEF | 2.027 | 1.911-2.150 | <0.0001 |

BMI: body mass index; MI: myocardial infarction, COPD: chronic obstructive pulmonary disease; EF: ejection fraction; HFrEF: Heart failure with reduced ejection fraction; HR: hazard ratio; CI: confidence interval.

*Left main disease was defined as an obstruction>50% in one of 2 major left circulation branches (left anterior descending or left circumflex coronary artery)

**Ventilation hours was assessed in a subgroup of patients that underwent on-pump surgery.

***Number of opium ever users, including former and current opium consumers.

Supplementary table 2. The univariate MACCE analysis.

|  | HR | 95% CI | P-value |
| --- | --- | --- | --- |
| Diabetes | 1.43 | 1.36-1.49 | <0.0001 |
| Age | 1.023 | 1.021-1.025 | <0.0001 |
| Male Gender | 0.819 | 0.780-0.860 | <0.0001 |
| BMI | 1.002 | 0.997-1.007 | 0.488 |
| Hypertension | 1.324 | 1.265-1.385 | <0.0001 |
| Dyslipidemia | 0.997 | 0.953-1.043 | 0.897 |
| Off-pump | 1.366 | 1.239-1.506 | <0.0001 |
| PreMI u7 | 1.149 | 1.057-1.249 | 0.001 |
| PreMI u24 | 1.125 | 0.983-1.288 | 0.086 |
| preCreatinine | 1.216 | 1.185-1.248 | <0.0001 |
| Family history | 0.863 | 0.824-0.904 | <0.0001 |
| Cigarette smoking: |  |  |  |
| 1-Current | 1.012 | 0.952-1.077 | 0.694 |
| 2-Former | 0.999 | 0.943-1.059 | 0.970 |
| Left main disease* | 1.149 | 1.058-1.248 | 0.001 |
| Graft number | 0.965 | 0.941-0.990 | 0.005 |
| Total ICU hours | 1.001 | 1.001-1.001 | <0.0001 |
| Total ventilation hours** | 1.001 | 1.001-1.001 | <0.0001 |
| Opium consumption*** | 1.109 | 1.042-1.180 | 0.001 |
| COPD | 1.438 | 1.295-1.598 | <0.0001 |
| Total EF | 0.976 | 0.974-0.979 | <0.0001 |
| HFrEF | 1.466 | 1.398-1.537 | <0.0001 |

BMI: body mass index; MI: myocardial infarction, COPD: chronic obstructive pulmonary disease; EF: ejection fraction; HFrEF: Heart failure with reduced ejection fraction; HR: hazard ratio; CI: confidence interval.

*Left main disease was defined as an obstruction>50% in one of 2 major left circulation branches (left anterior descending or left circumflex coronary artery)

**Ventilation hours was assessed in a subgroup of patients that underwent on-pump surgery.

***Number of opium ever users, including former and current opium consumers.

*Supplementary table 3. Independent effect of each variable on all-cause mortality after adjustment for the rest of the listed variables*

|  | HR | 95% CI | P-value |
| --- | --- | --- | --- |
| Diabetes | 1.516229 | 1.424326-1.614062 | <0.0001 |
| Age | 1.055233 | 1.051776-1.058701 | <0.0001 |
| Male Gender | 0.85262 | 0.789757-0.920486 | <0.0001 |
| BMI | 1.003426 | 0.995993-1.010914 | 0.367 |
| Hypertension | 1.273885 | 1.193605-1.359563 | <0.0001 |
| Dyslipidemia | 0.850651 | 0.797922-0.906865 | <0.0001 |
| Off-pump | 1.61788 | 1.414607-1.850363 | <0.0001 |
| PreMI u24 | 0.932565 | 0.777318-1.118818 | 0.452 |
| preCreatinine | 1.352263 | 1.312645-1.393078 | <0.0001 |
| Family history | 0.867957 | 0.813638-0.925903 | <0.0001 |
| Cigarette smoking: |  |  |  |
| 1-Current | 1.273443 | 1.160171-1.397774 | <0.0001 |
| 2-Former | 1.16545 | 1.072892-1.265994 | <0.0001 |
| Left main disease* | 1.161558 | 1.046241-1.289586 | 0.005 |
| Graft number | 0.977748 | 0.943313-1.013439 | 0.219 |
| Total ICU hours | 1.001549 | 1.001436-1.001662 | <0.0001 |
| Opium consumption** | 1.398681 | 1.280689-1.527544 | <0.0001 |
| COPD | 1.448812 | 1.271834-1.650416 | <0.0001 |
| Total EF | 0.957163 | 0.951821-0.962536 | <0.0001 |
| HFrEF | 0.870753 | 0.777803-0.974811 | 0.016 |

BMI: body mass index; MI: myocardial infarction, COPD: chronic obstructive pulmonary disease; EF: ejection fraction; HFrEF: Heart failure with reduced ejection fraction; HR: hazard ratio; CI: confidence interval.

*Left main disease was defined as an obstruction>50% in one of 2 major left circulation branches (left anterior descending or left circumflex coronary artery)

**Number of opium ever users, including former and current opium consumers.

Supplementary table 4. Independent effect of each variable on MACCE after adjustment for the rest of the listed variables

|  | HR | 95% CI | P-value |
| --- | --- | --- | --- |
| Diabetes | 1.313251 | 1.251008-1.378591 | <0.0001 |
| Age | 1.020871 | 1.018372-1.023375 | <0.0001 |
| Male Gender | 0.8116301 | 0.7652139-0.8608618 | <0.0001 |
| BMI | 1.005403 | 0.9997374-1.011102 | 0.062 |
| Hypertension | 1.19873 | 1.140294-1.260161 | <0.0001 |
| Dyslipidemia | 0.9508227 | 0.904993-0.9989725 | 0.045 |
| Off-pump | 1.302672 | 1.172742-1.446996 | <0.0001 |
| PreMI u24 | 0.9928794 | 0.8638361-1.1412 | 0.92 |
| preCreatinine | 1.232626 | 1.199647-1.266512 | <0.0001 |
| Family history | 0.9450568 | 0.9003069-0.992031 | 0.022 |
| Cigarette smoking: |  |  |  |
| 1-Current | 1.210845 | 1.127736-1.300079 | <0.0001 |
| 2-Former | 1.095924 | 1.027149-1.169304 | 0.006 |
| Left main disease* | 1.090793 | 1.002087-1.18735 | 0.045 |
| Graft number | 0.9692968 | 0.9431761-0.9961409 | 0.025 |
| Total ICU hours | 1.001453 | 1.001339-1.001567 | <0.0001 |
| Opium consumption** | 1.181693 | 1.102193-1.266927 | <0.0001 |
| COPD | 1.271958 | 1.140384-1.418712 | <0.0001 |
| Total EF | 0.9767603 | 0.9724725-0.9810669 | <0.0001 |
| HFrEF | 0.9383639 | 0.8583358-1.025853 | 0.162 |

BMI: body mass index; MI: myocardial infarction, COPD: chronic obstructive pulmonary disease; EF: ejection fraction; HFrEF: Heart failure with reduced ejection fraction; HR: hazard ratio; CI: confidence interval.

*Left main disease was defined as an obstruction>50% in one of 2 major left circulation branches (left anterior descending or left circumflex coronary artery)

**Number of opium ever users, including former and current opium consumers.
